# Research on application of multiparametric MRI to predict FNCLCC grading and ki67 expression in soft tissue sarcoma biopsy pathology: Based on a CT-MRI fusion image registration method

**DOI:** 10.3389/fonc.2025.1606942

**Published:** 2025-08-08

**Authors:** Nan Xu, Yang Lu, Xingrong Yang, Juan Tao, Lei Shi, Chuanshu Sun, Shaowu Wang

**Affiliations:** ^1^ Department of Radiology, Second Affiliated Hospital of Dalian Medical University, Dalian, China; ^2^ Department of Pathology, Second Affiliated Hospital of Dalian Medical University, Dalian, China; ^3^ Department of Radiology, Inner Mongolia Autonomous Region Hospital of Traditional Chinese Medicine, Hohhot, China

**Keywords:** CT-MRI fusion registration, precise imaging-pathology control, mp MRI, STS, CT-guided percutaneous CNBs, FNCLCC grading, ki67

## Abstract

**Objective:**

To investigate an *in vivo* biopsy method based on CT-MRI fusion image registration and evaluate its value for improving the accuracy of preoperative core needle biopsies(CNBs) histological grading in soft tissue sarcoma (STS)and determining the correlation between multiparametric MRI(mp MRI) and both STS French Federation of Cancer Center (FNCLCC) grading and the Ki-67 labeling index (LI).

**Methods:**

32 STS patients were enrolled prospectively and underwent 3.0T MRI, Diffusion weighted imaging(DWI) and Hydrogen proton magnetic resonance spectroscopy (^1^H MRS) examination, then underwent a preoperative CT guided CNBs that was confirmed by subsequent surgery. We used a novel image fusion registration method to ensure the biopsy sampling point was located in a high cell density area and the MRI-Region of Interest (ROI) was compatible with the biopsy specimen. We evaluated the accuracy of biopsy pathology diagnoses compared to that of surgery, and compared the mp MRI parameters between FNCLCC low- and high-grade/Ki67 low- and high-expression groups. The statistical analyses included the intraclass correlation coefficient(ICC), the Wilcoxon rank-sum test, Receiver Operating Characteristic (ROC) curves, and Spearman’s rank correlation.

**Result:**

The pathological diagnosis accuracy of the biopsy specimens was 100%, the subtype diagnosis accuracy was 87.5%, while the grading diagnosis accuracy was 93.8% compared with that of the surgical specimens. The FNCLCC high-grade group and Ki67 high-expression group had lower Apparent diffusion coefficient(ADC) values and higher Cholin/Creatin (Cho/Cr) ratios. The area under the curve(AUC) of ADCmin, ADCmean values and Cho/Cr ratios to discriminate between low- and high-grade groups were 0.956,0.969,0.917, low and high expression groups were 0.929,0.957,0.943; The ADC values of STSs correlated negatively while the Cho/Cr ratios correlated positively with FNCLCC grading (r=-0.782,-0.814, 0.758,p<0.001) and Ki-67 LI (r=-0.853, -0.902,0.710,p<0.001).

**Conclusion:**

The CT-MRI fusion image registration method may improve the accuracy of pre treatment STS CNBs pathology diagnoses and can be used for precise imaging-pathology control studies. ADC values and Cho/Cr ratios may therefore serve as a valuable supplement to STS histopathological grading and correlate with Ki67 expression.

## Introduction

STSs are a group of malignant tumors among soft tissue tumors with invasive or metastatic characteristics and exhibit high heterogeneity and diverse histological subtypes. Therefore, it is crucial to identify the histological subtype and pathological grade of STSs before treatment (1, 2). For low-grade sarcomas in the localized stage, surgical resection can be considered as a treatment option, while for high-grade sarcomas, neoadjuvant therapy is often recommended before surgery. Histological grading of STS is based on the FNCLCC system, which includes tumor characteristics such as mitotic rate, degree of differentiation, and necrosis. In addition, the Ki-67 index, as an independent prognostic factor for tumor prognosis (3), is also one of the key pathological markers that clinicians focus on before initiating STS treatment.

The diagnosis of STS prior to treatment is conducted primarily through biopsy. With the application of coaxial needles, the risk of needle tract seeding metastasis has been reduced significantly (4). However, compared to surgical specimens, the histological subtype and grading diagnosis of CNB specimens is not always accurate, with studies showing that the consistency of grading between pre-treatment CNBs and surgical resection specimens were from 32% to 87% (5, 6). The most likely reason for the low accuracy of biopsies is the high heterogeneity of STS tumors. Biopsy specimens may not accurately reflect all tumor information due to the poor resolution of soft tissue images guided by ultrasound or CT.

MRI is widely established as the preferred imaging modality for evaluating qualitative and quantitative features of STS. Qualitative MRI features, including peritumoral enhancement, T2-weighted image (T2WI) signal heterogeneity and necrotic area demonstrate significant correlation with FNCLCC grade (7–9). Serving as critical components of Imaging Cognitive Fusion methodology, these characteristics helps planning needle trajectories and puncture sites during Image guided CNBs (10). However, the correlation between qualitative MRI features of STS and histological grading faces significant influence from subjective factors, notably the interpreting radiologist’s image analysis proficiency and clinical expertise.

Several studies used quantitative parameters of functional MRI to evaluate the benignity/malignancy, histological grading, and expression of pathological markers in STS (11–15). However, determining the extent to which imaging reflects pathological changes, most research methods directly compare the imaging features of tumors with the results of their histological or cytological pathological tests. However, this is not an accurate match between the ROI of the image and the pathological sampling point and may lead to deviations in the pathological interpretation of the image.

Yuan et al. (16) established a nude mouse model of rhabdomyosarcoma and evaluated the relationship between fMRI quantitative parameters and pathological indicators of rhabdomyosarcoma using a new and precise method to compare imaging pathology. They showed that DWI and intravoxel incoherent motion (IVIM) parameters were helpful for accurately evaluating Ki-67 expression levels. Subsequently, they applied this method to clinical research and investigated the accuracy of the Dynamic contrast enhanced Magnetic resonance imaging (DCE-MRI), IVIM, and Diffusion kurtosis imaging (DKI) parameters to predict STS pathological grading and Ki-67 expression. That study further confirmed the method achieved an accurate comparison between imaging and surgical specimen pathology of superficial soft tissue masses in limbs (17–20). However, this method is only applicable to surgical specimens, whereas a considerable number of sarcoma patients have lost the opportunity for surgery or require neoadjuvant therapy before undergoing surgery. This group of patients therefore require a method for comparing imaging and pathology using biopsy specimens in order to improve the accuracy of biopsies and also precise comparative studies between imaging features and pathological indicators of biopsy specimens.

This study used a novel image registration method based on *in vivo* CNBs that involved enhanced MRI and DWI to select and label the CNBs target slice, thereby enabling surgeons to puncture the lesion area with cellular activity. The MRI image of the target slice is then fused and registered with the CT image of the needle tip slice to delineate the ROI, thereby achieving accurate matching and comparison of “MRI ROI pathology sampling points”. The study also included a preliminarily investigation of the correlation between mp MRI parameters and pathological markers.

## Materials and methods

The local institutional ethics board approved the prospective study design(Consent Letter Number: 2022053 and KY2025-69-01). Informed consent was obtained from all the patients.

### Patients

**Figure 1:**
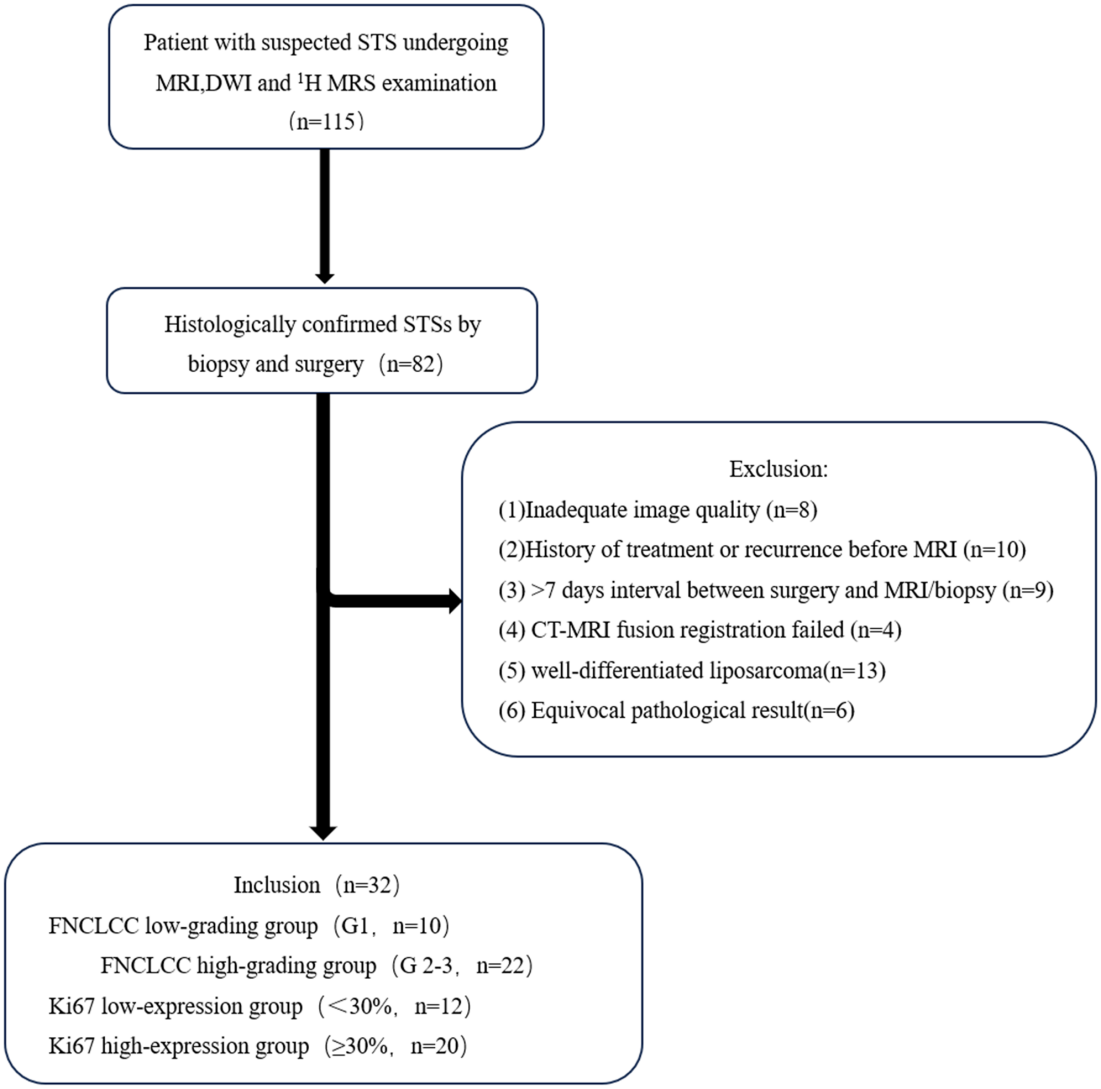
Study flowchart.

From June 2022 to October 2024, 115 patients suspected of having a STS underwent enhanced MRI and DWI examinations, followed immediately by CT guided core needle biopsies (CNBs). A total of 32 cases were included and 83 cases were excluded. The exclusion criteria were: (1) Inadequate MRI image quality(n=8); (2) history of treatment or recurrence before the MR examination(n=10); (3) >7 days interval between surgery and the MRI/CNBs (n=9); (4) failed CT-MRI fusion registration (n=4); (5) well-differentiated liposarcoma(as the adipose tissue components demonstrate signal suppression on DWI, which precludes accurate measurement of ADCvalues) (n=13); and (6) an equivocal pathological result (n=6). The flow chart of the study is shown in **Figure 1**.

### Imaging data

All the MRI examinations were performed on a 3.0T scanner (Discovery MR750w, GE Healthcare) with a 16-channel phased array body coil depending on the tumor location, equipped with an ADW4.6 workstation and Functool software. A conventional MRI was performed using a fast spin-echo sequence, including T1-weighted imaging (T1WI) Axial, T1WI fat suppressed (T1WI-FS)Axial, T2WI Axial, T2WI fat suppressed (T2WI-FS)Axial, T1WI-FS Axial C+,DWI and ^1^H MRS. DWI was performed before the enhancement scan, using a spin-echo echo-planar imaging (SE-EPI) sequence with the following parameters: repetition time (TR)=3000–3711 ms, echo time (TE)=68–70 ms, and matrix size=256 × 224. The field of view (FOV), slice thickness, and slice spacing were matched to those of the axial T2-weighted imaging (T2WI) sequence. DWI was obtained with diffusion sensitivity coefficients (b-values) of 0 and 800 s/mm², and the number of excitations (NEX) was set to 4. ^1^H MRS was performed after DWI examination, using a PROBE-P sequence and multi voxel imaging scan with the following parameters: TR=1000ms, TE=144ms, matrix size=128×128,and NEX=1.Saturation bands were employed to shield interfering signals such as water, bone, blood vessels, and nerves around the lesion, with a voxel size of 2 × 2 × 2 mm. CT-guided CNB was performed on a 64-slice CT scanner(Siemens Sensation) after DWI examination with the following parameters: tube voltage=120kV, tube current=200mAs, pitch=0.9mm, rotation time=0.6s, and matrix size=512 × 512.Other CT scan data (position, FOV, slice thickness, and slice gap) were completely consistent with the MRI scan data.

All the MRI raw images were processed using the Advantage Workstation (ADW 4.7, GE Healthcare) software to generate corresponding pseudo-color images and measure the quantitative parameters. ROI selection: two radiologists (10 years of CNBs experience and 12 years of musculo-skeletal imaging experience, respectively) blinded to the patients’ histopathological results independently placed ROI on the CT-MRI fusion image. A 2D rectangular ROI measuring 2cm×0.2cm was positioned anterior to the needle tip, aligned with the puncture trajectory to approximate the shape of the biopsy specimen. For statistical analysis, the mean values of ROI measurements were utilized.

### CT-MRI image fusion registration method

**Figure 2:**
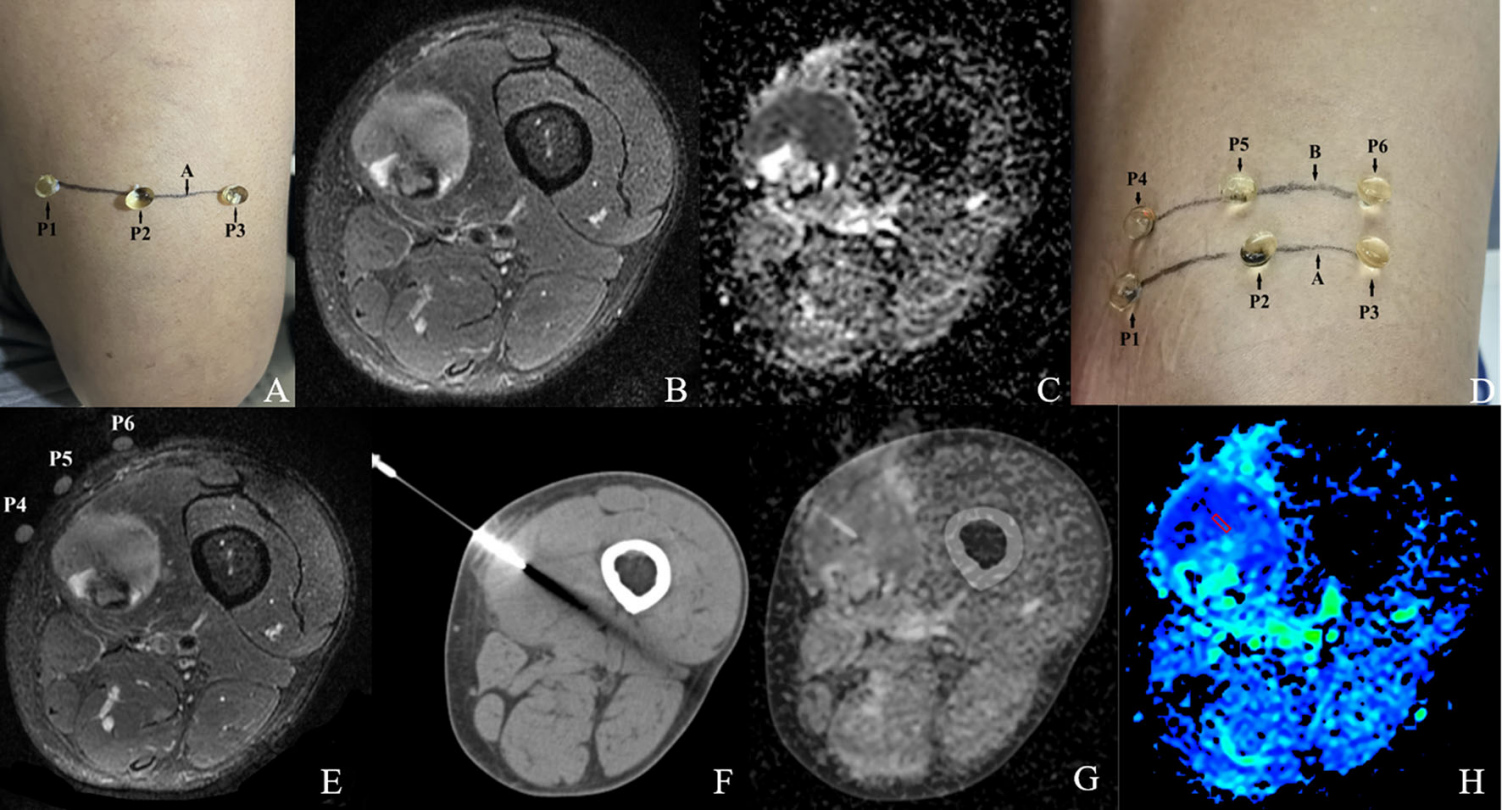
CT-MRI image fusion registration method.

We evaluated the CT-MRI image fusion registration method based on an *in vivo* CT-guided percutaneous CNB, with the aim of establishing a MRI-pathology control. This method is described in **Figure 2**.

Step 1: Before MRI scanning, the general location of the tumor lesion was determined by ultrasound or palpation, and a horizontal line A drawn over the mass at the most palpable site. Three vitamin D capsule markers were positioned on line A as P1, P2 and P3 (**Figure 2A**).

Step 2: The 3.0 T MRI scanning protocol included T2WI-FS and DWI sequences. The patient’s position, FOV value, slice thickness, and slice gap were recorded (lesion with a head-to-foot size <5 cm; slice thickness 3 mm, slice gap 1 mm; lesion with a head-to-foot size ≥5 cm; slice thickness 5 mm, slice gap 1 mm) (**Figures 2B, C**).

Step 3: The slice with a large area of hyperintensity on T2WI-FS, hyperintensity on DWI while hypointensity on ADC was selected jointly as the target slice by the two radiologists mentioned earlier. This was calculated as the distance S between the capsule slice and the target slice (S= (slice thickness + slice gap)×slice number). A horizontal line B was then drawn over the mass using data S to mark the target slice. Another three vitamin D capsules markers were positioned on line B as P4, P5, and P6. A further MRI scan was performed to confirm whether the capsule (P4-6) slice overlapped with the target slice. Once consistency was confirmed we completed all the MRI scans. If the signal characteristics of the capsule (P1-3) slice matched that of the target slice there was no need to draw line B or carry out a repeat scan (**Figures 2D, E**).

Step 4: CT-guided CNB was performed immediately after the MRI scan using a 15-gauge Coaxial needle with a 16-gauge biopsy needle(0.16cm diameter). The CT scan data (position, FOV, slice thickness, and slice gap) were completely consistent with the MRI scan data. The puncture point was selected at the line B level of the body surface, (i.e. the target slice) so that the coaxial needle was perpendicular to line B and inserted into the target slice of the lesion. After confirming the position, the biopsy specimen (size 2×0.2 cm) was obtained (**Figure 2F**).

Step 5: The MR image of the target slice and the CT image of the needle tip were processed using the Advantage Workstation (Syngo.via, Siemens) for Auto Calibration and fusion (21). Markers such as capsules P4-6, bones, and blood vessels were used for manual fusion if the automatic fusion was not satisfactory. The two radiologists mentioned earlier jointly evaluated the quality of the fused image. The ROI selection method was as mentioned above (**Figures 2G, H**).

### Pathological analysis

In accordance with the 2020 WHO classification of STS, the histopathologic results of all the biopsy specimens and surgical specimens were diagnosed independently by two pathologists with over 10 years of experience in musculo-skeletal pathology who were blinded to the clinical and imaging data. Similar to previous publications, grade II/III STSs were classified as high-grade STS and grade I STS as low-grade STS (18, 22). Ki-67 positivity was determined when the nuclei displayed brown-yellow coloration. Only the areas with the highest number of positive cells (hot spots) were analyzed. Ki-67LI was evaluated using the percentage of positive cells from 1000 tumor cells (× 400). The Ki-67 levels were divided into low (< 30%) and high (≥ 30%) Ki-67 expression groups (20).

### Statistical analysis

All the data were analyzed using SPSS 24.0 (IBM). The Shapiro-Wilk test was employed to assess the normality of all continuous variable data. Data conforming to a normal distribution were presented as mean ± standard deviation, while non-normally distributed data were expressed as median (P25, P75). Inter-reader reliability between the two observers was evaluated using the intraclass correlation coefficient (ICC), with an ICC value >0.75 indicating good agreement. The independent samples t-tests or Wilcoxon rank-sum tests were used to detect differences in the ADC and ^1^H MRS parameters between the FNCLCC low and high graded groups and Ki-67 low and high expression groups in the STSs. Spearman’s rank correlation was used to analyze the association between ADC and ^1^H MRS parameters, FNCLCC grading, and Ki-67LI. A receiver operating characteristic (ROC) curve was prepared for each parameter, with the area under the curve (AUC) analyzed to evaluate diagnostic performance. The optimal diagnostic cut-off value and corresponding sensitivity and specificity were obtained according to the Youden index for each parameter and Ki-67 LI. A p value < 0.05 was considered statistically significant.

## Results

### Patient characteristics and CNBs pathological diagnoses

Of the 32 patients, 18 were males and 14 were females with a mean age of 53.67 ± 3.81 years. The lesions were located in 12 upper limbs, 16 lower limbs, and 4 in the trunk, with a maximum diameter of 6.2 ± 2.5 cm. We additionally quantified the MRI scanning duration during the second stage of CT-MRI image registration, recording an average scan time of 7 minutes 32 seconds ±46 seconds. This represents a moderate temporal increase compared to conventional MRI protocols. Based on the results of the pathological diagnoses of the surgical specimens, all 32 CNBs pathological diagnoses were sarcomas (accuracy rate, 100%). 28 pathological subtypes matched the surgical specimens, while 2 subtypes were not diagnosed (both myxoid sarcomas) and 2 subtypes were diagnosed incorrectly (accuracy rate, 87.5%). In terms of grading; low/high grade (8/24) were diagnosed by CNBs and low/high grade (10/22) were diagnosed by surgery (accuracy rate, 93.8%). For Ki-67 LI, 12 patients were classified in the low expression group and 20 patients in the high expression group. The histological subtypes, FNCLCC grading, and Ki-67 expression results of all the patients are shown in **Table 1**.

**Table 1:** The histological subtypes, FNCLCC grading, and Ki67 expression results.

Pathological Parameters	Variable	n
Histologic subtype (surgery)	Dedifferentiated liposarcoma	2
	Myxoid liposarcoma	3
	Pleomorphic liposarcoma	1
	leiomyosarcoma	8
	synovial sarcoma	2
	fibrosarcoma	6
	undifferentiated pleomorphic sarcoma	2
	Malignant tenosynovial giant cell tumor	1
	Extraskeletal Ewing sarcoma	1
	solitary fibrous tumor	1
	angiosarcoma	1
	alveolar soft part sarcoma	2
	rhabdomyosarcoma	2
FNCLCC Grade (biopsy/surgery)	Low grade(I)	8/10
	High grade(II/III)	24/22
Ki67 LI (biopsy)	Low expression	12
	High expression	20

### Correlation of DWI,^1^H MRS parameters and FNCLCC grading, Ki67 LI in the biopsy specimens

As shown in **Tables 2**, The Ki-67 LI and Cho/Cr values of all 32 patients followed a normal distribution, while both ADCmin and ADCmean values exhibited non-normal distribution patterns. The interobserver agreement analysis revealed excellent reproducibility, with ICC ranging from 0.604 to 0.968.

**Table 2:** Measurements of Ki-67 LI and MRI parameters and inter-reader ICC.

Parameters	N	Range	Mean ± SD / median (P25, p75)	ICC	95%CI	*P*
Ki-67 KI	32	5-90%	46.2±26.0%	0.938	0.895-0.966	<0.001
ADCmean(×10^-3^mm^2^/s)	32	0.81-3.38	1.22(1.08,1.66)	0.867	0.782-0.949	<0.001
ADCmin(×10^-3^mm^2^/s)	32	0.53-2.91	1.02(0.85,1.21)	0.883	0.795-0.968	<0.001
Cho/Cr	32	0.65-13.14	1.03±0.88	0.807	0.604-0.912	<0.001

**Figure 3:**
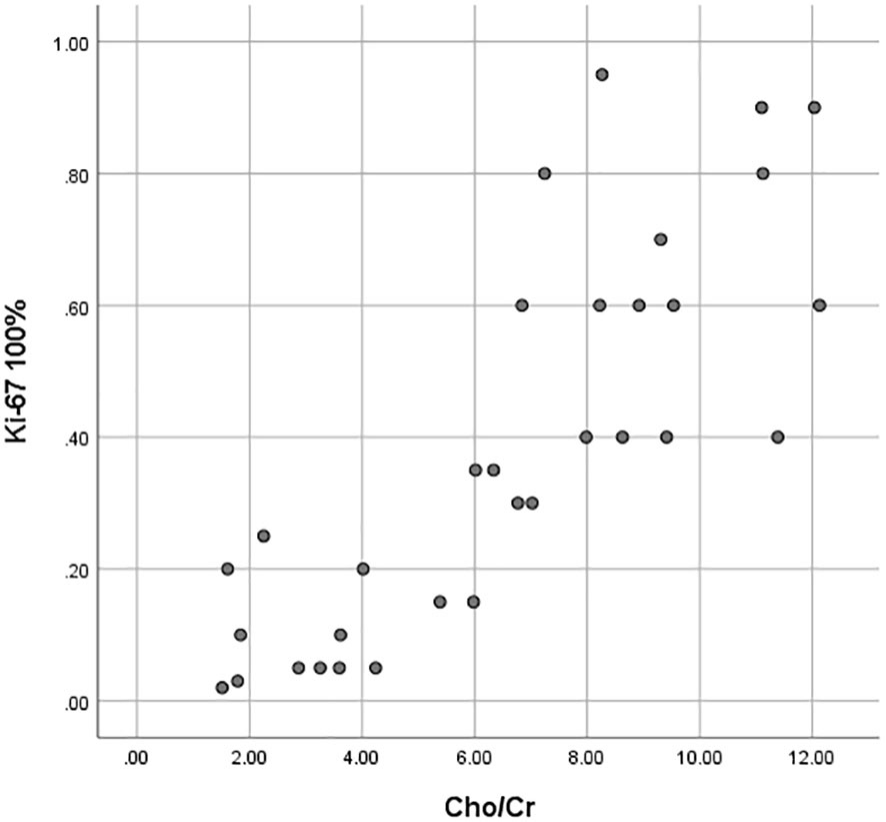
Scatter plots of the correlation between the Ki-67 LI with ADCmin, ADCmean values and Cho/Cr ratios.

As shown in **Tables 3**, **4** and **Figure 3**, the values of ADCmin and ADCmean were significantly lower, while the Cho/Cr ratios were higher in the FNCLCC high grade and Ki67 high expression groups, compared to those in the low grade and low expression groups. ADCmin, ADCmean values correlated negatively, while Cho/Cr ratios correlated positively with FNCLCC Grading and Ki-67 LI; correlation coefficients were −0.782,−0.814,0.758 and−0.853,-0.902,0.710,respectively (p < 0.001),with the correlation with ADCmean values being stronger.

**Table 3:** Comparison of DWI and ^1^H MRS parameters of STSs in FNCLCC grading and Ki-67 LI.

Parameters	FNCLCC grading				Ki-67 LI			
	High grade group	Low grade group	*Z/t*	*p*	High expression group	Low expression group	*Z/t*	*p*
ADCmin (×10^-3^mm^2^/s)	0.93 (0.81,1.06)	1.33 (1.24,2.49)	-2.380	0.017	0.91 (0.78,1.03)	1.32 (1.15,1.38)	-3.061	0.002
ADCmean (×10^-3^mm^2^/s)	1.16 (1.04,1.29)	1.63 (1.59,2.78)	-2.521	0.012	1.12 (0.97,1.22)	1.61 (1.50,1.66)	-2.981	0.003
Cho/Cr	10.24±2.61	4.43±1.95	7.282	<0.001	13.98±3.17	7.05±3.28	8.908	<0.001

**Table 4:** Correlation of DWI and ^1^H MRS parameters of STSs in FNCLCC grading and Ki-67 LI.

Parameters	FNCLCC grading		Ki-67 LI	
	r	*p*	r	*p*
ADCmin(×10^-3^mm^2^/s)	-0.782	<0.001	-0.853	<0.001
ADCmean(×10^-3^mm^2^/s)	-0.814	<0.001	-0.902	<0.001
Cho/Cr	0.758	<0.001	0.710	<0.001

**Figure 4:**
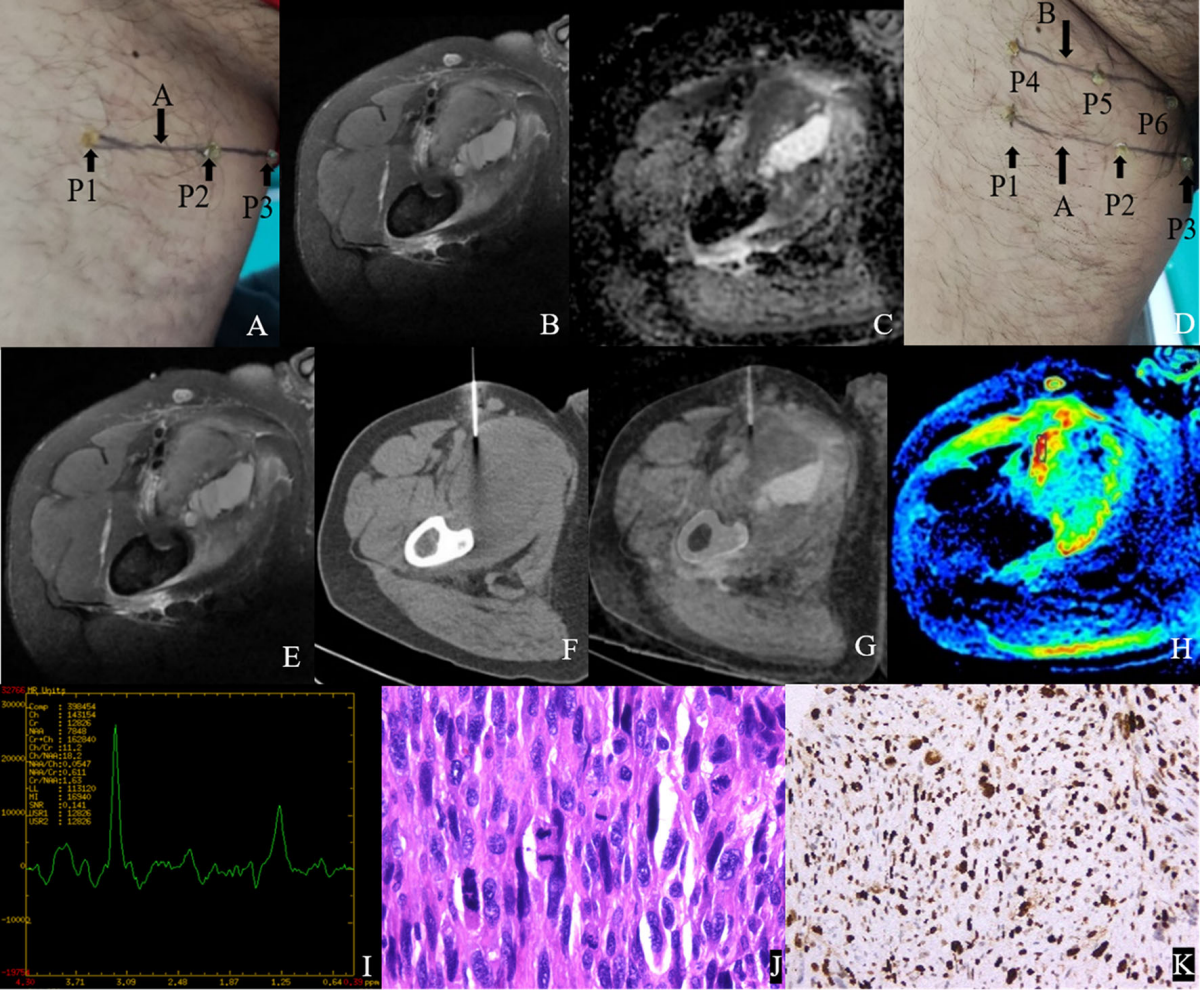
Male, 47Y, Pleomorphic leiomyosarcoma of the right thigh, G3. **(A)** Body surface positioning marks before MRI examination; **(B, C)** Targeted slice, chosen by Axial T2WI-FS and ADC image; **(D)** Mark the targeted slice on body surface; **(E)** A second time Axial T2WI-FS scan to confirm the target slice; **(F)** CT guided biopsy at target slice; **(G)** CT-MRI image fusion at target slice; **(H)** Draw ROI to obtain DWI quantitative parameters, ADCmin: 0.725×10–^3^ mm^2^/s, ADCmean: 0.929×10–^3^ mm^2^/s; **(I)** ^1^H MR spectrum, Cho/Cr:11.2; **(J)** Histological images of biopsy specimens, Grade 3, HE×200; **(K)** Ki67 index 70%.

**Figure 5:**
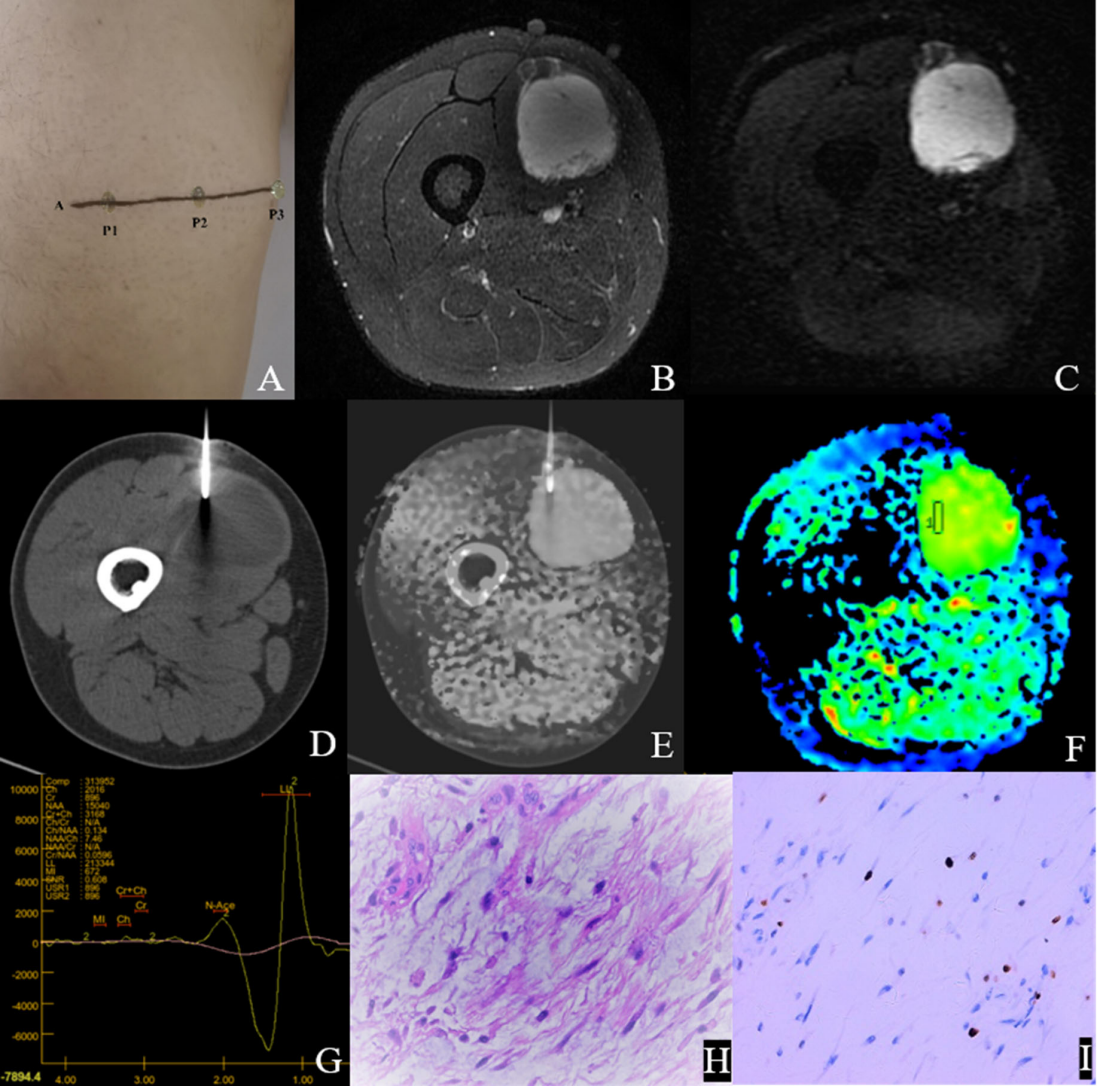
Male,35Y, Myxofibrosarcoma of the right calf, G1. **(A)** Body surface positioning marks before MRI examination; **(B, C)** Targeted slice, chosen by Axial T2WI-FS and ADC image; **(D)** CT guided biopsy at target slice; **(E)** CT-MRI image fusion at target slice; **(F)** Draw ROI to obtain DWI quantitative parameters, ADCmin: 3.280×10–^3^ mm^2^/s, ADCmean: 3.370×10–^3^ mm^2^/s; **(G)** ^1^H MR spectrum, Cho/Cr:2.25; **(H)** Histological images of biopsy specimens, Grade3, HE×200; **(I)** Ki67 index 10%.

**Figures 4** and **5** show the features of the CT-MR fusion registration images, pathological diagnosis, and Ki-67 LI images of the histologically confirmed pleomorphic leiomyosarcomas and myxofibrosarcomas.

### Diagnostic efficacy analysis of DWI and ^1^H MRS parameters for FNCLCC grading and Ki67 expression

**Figure 6:**
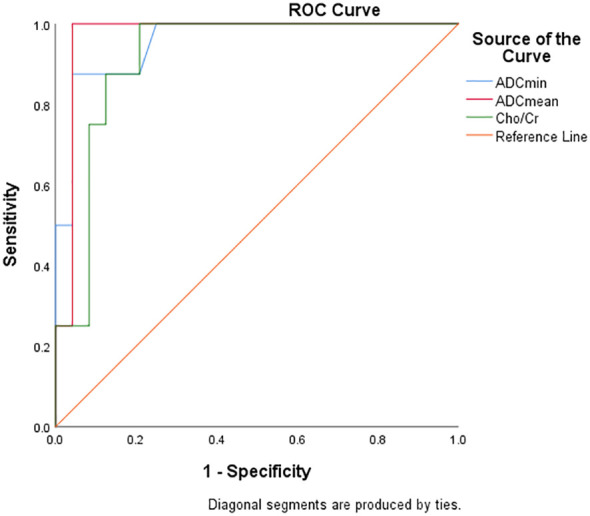
ROC curve showed diagnostic performance of ADCmin, ADCmean and Cho/Cr in FNCLCC grading.

**Figure 7:**
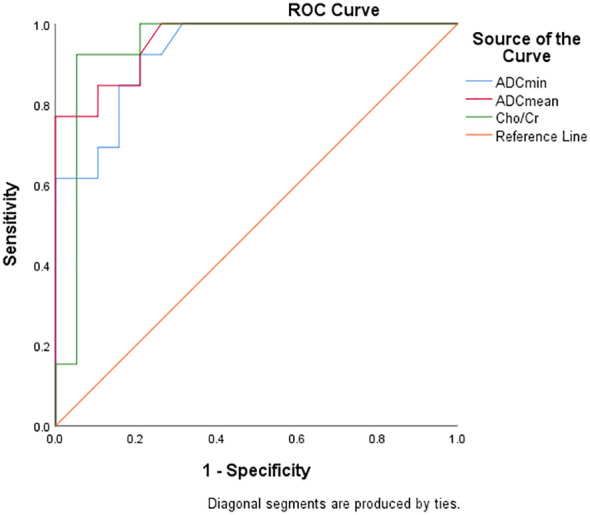
ROC curve showed diagnostic performance of ADCmin, ADCmean and Cho/Cr in Ki67 LI.

**Table 5** and **Figure 6** show the diagnostic efficiency of the DWI and ^1^H MRS parameters for differentiating between the low and high FNCLCC grade groups, while **Table 6** and **Figure 7** show the diagnostic efficiency of DWI and ^1^H MRS parameters for differentiating between the low and high Ki-67 expression groups. In both ROC analyses, ADCmean had a higher AUC (0.969, 0.957). The optimal cut-off ADCmean value of 1.525 was associated with 100% sensitivity and 95.8% specificity in the high grade group. In the high expression group, the value was 1.450 with 76.9% sensitivity and 100.0% specificity.

**Table 5:** Diagnostic performance of DWI and ^1^H MRS parameters for differentiating low and high grade groups of FNCLCC.

Parameters	AUC	95% CI	P	Cutoff value	Sensitivity (%)	Specificity (%)	Youden index
ADCmin	0.956	0.886-1.000	<0.001	<1.170	87.5	95.8	0.833
ADCmean	0.969	0.906-1.000	<0.001	<1.525	100.0	95.8	0.958
Cho/Cr	0.917	0.828-1.000	<0.001	>5.910	79.2	100	0.792

**Table 6:** Diagnostic performance of DWI and ^1^H MRS parameters for differentiating low and high expression groups of Ki-67 in STS.

Parameters	AUC	95% CI	P	Cutoff value	Sensitivity (%)	Specificity (%)	Youden index
ADCmin	0.929	0.846-1.000	<0.001	<0.935	92.3	78.9	0.713
ADCmean	0.957	0.896-1.000	<0.001	<1.450	76.9	100	0.769
Cho/Cr	0.943	0.853-1.000	<0.001	>5.997	94.7	92.3	0.870

## Discussion

The key step and challenge for accurately determining tumor pathological changes using imaging is to ensure precise matching between the imaging ROI and histopathological slices. Currently, such research approaches have been more extensively documented in prostate (23, 24) and brain (25–27), with clinical applications encompassing multiple domains including benign-malignant lesion differentiation, radiological-pathological correlation analysis, image-guided CNBs procedures, and intraoperative navigation systems. However, due to the fixed position of brain and prostate, registration is relatively simple but is not applicable to other tissues. Breast can also be regarded as a kind of soft tissue organ, which registration method is similar to that of soft tissue tumors. Maris et al. (28) registered high-resolution PET-CT images of *ex vivo* breast cancer specimens with digital whole slide images (WSI) using an automatic co-registration algorithm to precisely observe the standardized uptake values (SUVs) of different types of breast tumors. In soft tissue tumors, Jung et al. (29) successfully established a spatially exact co-registration procedure between *in vivo* MRI, *ex vivo* MRI and histopathology of STS to identify imaging parameters that reflect radiation therapy response of STS. Wang et al. adopted a “body position line-MRI slice-pathological section control method” (16–20), which also achieved accurate imaging-pathologic control of limb superficial soft tissue tumors.

However, many sarcoma patients loose the opportunity for surgery or require neoadjuvant therapy before undergoing surgery. Clinically, the FNCLCC grading diagnoses before treatment is usually based on biopsy pathology rather than surgical pathology. Therefore, it is equally important to improve the accuracy of CNB and the imaging-pathologic control of biopsy specimens. The imaging-pathologic control of previous biopsy specimens was performed using postoperative *ex vivo* biopsy specimens. As reported by Rasmussen et al. (30), MRI scans of 31 patients with head and neck squamous cell carcinoma were performed on preoperative and postoperative *in vitro* specimens. The tumor pathological specimens were formed into digital H&E images and fused with PET/MR images for registration. A CNBs was then performed on the *in vitro* specimens to accurately determine the correlation between the imaging parameters and tumor pathological markers. Hettler et al. (31) performed pre-operative *in vivo* MRI scans on 12 patients with STSs and then performed *ex vivo* CT scans and CNBs of post-operative specimens. Using Eclipse software, the pre-operative MRI images of the lesion and the post-operative *ex vivo* CT images were fused and imaged, and achieved precise comparison between the MRI ROI and biopsy sampling area. However, *ex vivo* specimens are soft and prone to deformation, and the extent to which ex vivo specimens can reliably represent the *in vivo* biological behavior of tumors remains a subject of ongoing scientific debate.

Although it was once envisioned in Hettler’s research (31), our study is the first to use *in vivo* MRI scanning of STS and CT-guided *in vivo* CNBs and integration of the MRI imaging slice with the CT-guided CNB sampling slice for image fusion and registration. The biopsy sampling point serves as the pathological sampling point, thereby eliminating the issues that arise after specimens are removed from the body. Secondly, this method essentially achieves precise imaging-pathologic control of biopsy specimens, and also not complicated. It is beneficial to establish a close logical connection between the imaging features of MRI and the pathological information of the specimen. This approach provides a precise “MRI ROI-histopathological slices” correlation method for predicting pathological changes in a STS, and therefore aids in accurate prediction of the STS diagnosis and prognosis. Thirdly, this method enables pathological diagnosis of biopsy specimens to approximate that of surgical specimens, thereby assisting surgeons to evaluate STSs more accurately before surgery.

Ultrasound-guided CNBs are used more commonly for soft tissue tumors and studies have reported an accuracy rate of up to 92.5% (32). However, the spatial resolution of ultrasound images is low, the slices are not fixed, and the image morphology are quite different from MRI, which makes it difficult to achieve precise matching with soft tissue MRI images, except for relatively fixed organs such as the prostate (33). Noebauer-Huhmann et al. (34) achieved reliable grading diagnoses in 90.5% of cases using MRI-guided CNBs for soft tissue tumors, although this method is difficult to implement widely in clinical practice. Therefore, in our study we chose to use CT-guided biopsies. In comparison to standard MRI protocols, the CT-MRI image registration process necessitates additional T2WI-FS and DWI sequences. Based on lesion size, this protocol extends the average MRI scanning time by approximately 7 minutes. Given the substantial diagnostic benefits of this approach, we consider this marginal time increase clinically acceptable and believe the methodology demonstrates strong feasibility for routine clinical implementation.

We selected the ADC value, one of the most commonly used quantitative parameters in MRI clinical examinations, as the imaging biomarker for STS. This was because DWI reflects the degree of water molecule diffusion in tissues, which correlates positively with cell density. Studies have shown that the ADC value has unique value for differentiating between benign and malignant soft tissue tumors and evaluating early prognosis, and decreases with increasing malignancy of STS (35–38). In this preliminary study, the results obtained being consistent with those of previous studies. We also selected the ^1^H MRS metabolite Cho based on its fundamental role in phospholipid metabolism, a critical component of cell membrane biosynthesis and degradation, which reflected the renewal of cell membranes and cell proliferation. Due to the relatively stable Cr value, Cho/Cr ratio is usually used as a metabolic marker in spectroscopic studies (39). Russo et al. (40) conducted a analysis of 43 STSs, revealing a significant positive correlation (p<0.01) between ^1^H-MRS Cho peak and mitotic index stratification. Similarly, Patni et al. (39) found that the Cho/Cr ratio was positively correlated with the histological grading of musculoskeletal tumors. However, the above retrospective studies were lack of Imaging-Pathologic correlation analysis. Besides, the inclusion of heterogeneous tumor types (particularly malignant bone tumors) might confound metabolic interpretations. In contrast to previous work, our prospective study implemented rigorous methodological controls to enhance diagnostic specificity. The results showed that Cho/Cr ratio exhibited a great diagnostic performance in evaluating STS histological grading and Ki-67 expression. Well-differentiated liposarcomas were systematically excluded due to the confounding effects of lipid signal suppression in DWI sequences, which could also significantly degrade spectral quality, thereby affected the accuracy of data measurement. However, the data in this study exhibited two outliers, resulting in a non normal distribution of ADC data and unstable spectral curves in these two cases. These outliers correspond to two cases of low-grade myxosarcoma characterized by minimal solid components. Higher ADC values in myxoid tissues make it unable to differentiate between benign and malignant myxoid soft tissue tumors (41). This histological feature also explains why biopsy specimens from these cases failed to yield a definitive histological subtype diagnosis. Gimber et al. reported trends toward more heterogeneous T1- and T2-weighted signal intensity in high-grade tumors within their series of 31 myxoid liposarcomas (42). Research shows that MRI radiomics utilizing T1-weighted, fat-suppressed T2-weighted, and contrast-enhanced T1-weighted sequences have demonstrated high diagnostic accuracy in differentiating benign myxomas from malignant myxoid sarcomas (43). For mucus-rich soft tissue tumors, further investigation with larger sample sizes and more parameters such as T1,T2 mapping or radiomics are required to validate and improve CNBs methodologies.

The FNCLCC grading and KI67 index are important criteria for developing treatment plans for STS patients. However, FNCLCC grading is based on biopsy specimens and is often an unreliable parameter in pre-treatment evaluation. The pathological diagnosis of surgical specimens is also inaccurate, as the application of preoperative treatment may significantly alter the FNCLCC and Ki67 parameters. Therefore, improving the accuracy of grading and Ki67 expression in pre-treatment biopsies is crucial for clinical treatment decisions. Studies have shown that replacing mitotic counts with Ki-67 enhances the accuracy of FNCLCC grading in STS (44). Given that functional imaging reflects tumor aggressiveness and heterogeneity by assessing tumor cell density and proliferative capacity, studies have examined the correlation between STS functional imaging parameters and KI67 index expression (18, 20, 45). Based on the findings of these studies we chose to use Ki67 in the current study.

Although the National Comprehensive Cancer Network(NCCN) Soft Tissue Sarcoma Diagnosis and Treatment Guidelines (2022) recommend image-guided biopsy as the preferred method for definitive pathological diagnosis before treatment (46). Radiomics has become a valuable tool for non-invasive prediction of STS histological grading and benign/malignant distinction (47, 48), which is expected to replace the role of biopsy in the future. The focus of the development of radiomics is on how to achieve trustful model, increasing open-science, increasing prospective multicentric trials, and building large databases to independently validate radiomics models. In addition, the implementation of deep learning and explanatory approaches involving transcriptomics data pave the way for a very exciting and challenging future for radiomics in STS patients.

This pilot study had some limitations. First, our small patient population, uneven distribution of anatomical sites of tumors and some specific STS subtypes(e.g. Alveolar Soft-Part Sarcoma) that do not follow FNLCC grading system may have led to selection bias. Considering this is a preliminary study and the low incidence rate of sarcoma, further improvement would be achieved in larger cohort studies that excludes certain sarcomas that do not follow FNLCC grading system (e.g. Alveolar Soft-Part Sarcoma, Myxoid Round Cell Liposarcomas, Clear Cell Sarcomas). Secondly, due to the lack of specialized sarcoma image fusion software, the study used commercial software in the Siemens post-processing system which did not calculate registration errors and may also have introduced bias. Thirdly, the CNBs path we chose should have avoided passing through multiple compartments to prevent metastasis, but may have affected the patient’s position in the CT scan, resulting in inconsistency of the position of the MR scan. This was the reason why we had four failed cases of fusion image registration. Fourthly, While ^1^H-MRS demonstrates significant potential in stratifying Fnclcc grading and quantifying Ki-67 proliferative activity in STS, the spectral instability observed in our cohort(n=8), thereby limiting the final sample size. Further research on how to control the quality of ^1^H MRS spectral lines in STS is necessary. In addition, our study attempted to use vitamin capsules to label and locate the target slice, which also served as an image registration marker and achieved good results.

## Conclusion

This study conducted a preliminary investigation of a fusion registration method between pre CNBs MRI images and *in vivo* CNBs CT images of STSs, and achieved a precise comparison of “MRI- ROI to biopsy histopathological slices”. Considering that pre-treatment grading is the basis of STS treatment plans, we demonstrated that CT guided CNBs using this method had a high accuracy in histopathological diagnosis, and that DWI and ^1^H MRS parameters accurately assessed STS Fnclcc grading and ki67 expression under precise imaging-pathology control. In the future, we plan to conduct larger cohort studies to confirm whether this method can be extended to more MRI functional sequences and other tumors, which may help to identify the best imaging biomarkers that reflect the biological behavior of tumors in various systems.

## Data Availability

The raw data supporting the conclusions of this article will be made available by the authors, without undue reservation.
